# Model-based design of the first steps of a seed train for cell culture processes

**DOI:** 10.1186/1753-6561-7-S6-P11

**Published:** 2013-12-04

**Authors:** Simon Kern, Oscar B Platas, Martin Schaletzky, Volker Sandig, Björn Frahm, Ralf Pörtner

**Affiliations:** 1 Institute of Bioprocess and Biosystems Engineering, Hamburg University of Technology, Hamburg, D-21073, Germany; 2 Biotechnology & Bioprocess Engineering, Ostwestfalen-Lippe University of Applied Sciences, Lemgo, D-32657, Germany; 3 ProBioGen AG, Berlin, D-13086, Germany

## Concept

Production of biopharmaceuticals for diagnostic and therapeutic applications with suspension cells in bioreactors requires a seed train up to production scale [1]. For the final process steps in pilot and production scale the scale-up steps are usually defined (e.g. a factor of 5 - 10). More difficult in this respect are the first steps, the transitions between T-flasks, spinner tubes, roller bottles, shake flasks, stirred bioreactors or single-use reactors, because here often scale-up steps are different. The experimental effort to lay these steps out is correspondingly high. At the same time it is known that the first cultivation steps have a significant impact on the success or failure on production scale. The concept for a model based design of the seed train consists of the following steps:

➢ A simple unstructured kinetic model, where kinetic parameters can be obtained from a few experiments only.

➢ A Nelder-Mead-algorithm to determine model parameters.

➢ A MATLAB simulation based on this model to determine optimal points in time or viable cell concentrations respectively for harvest of seed train scales from spinner tubes over shake flasks up to a stirred bioreactor based on an optimization criterion.

## Verification

The concept was verified for a suspendable cell line (AGE1.HN, ProBioGen AG) grown in serum-free 42-Max-UB medium (Teutocell AG, Germany) containing 5 mM-Glutamine.

Two batch experiments were performed in shake flasks for determination of kinetic parameters.

The average value of time for minimal and maximal Space-Time-Yield for cells was used as optimization criterion for cell transfer.

**Figure 1:**
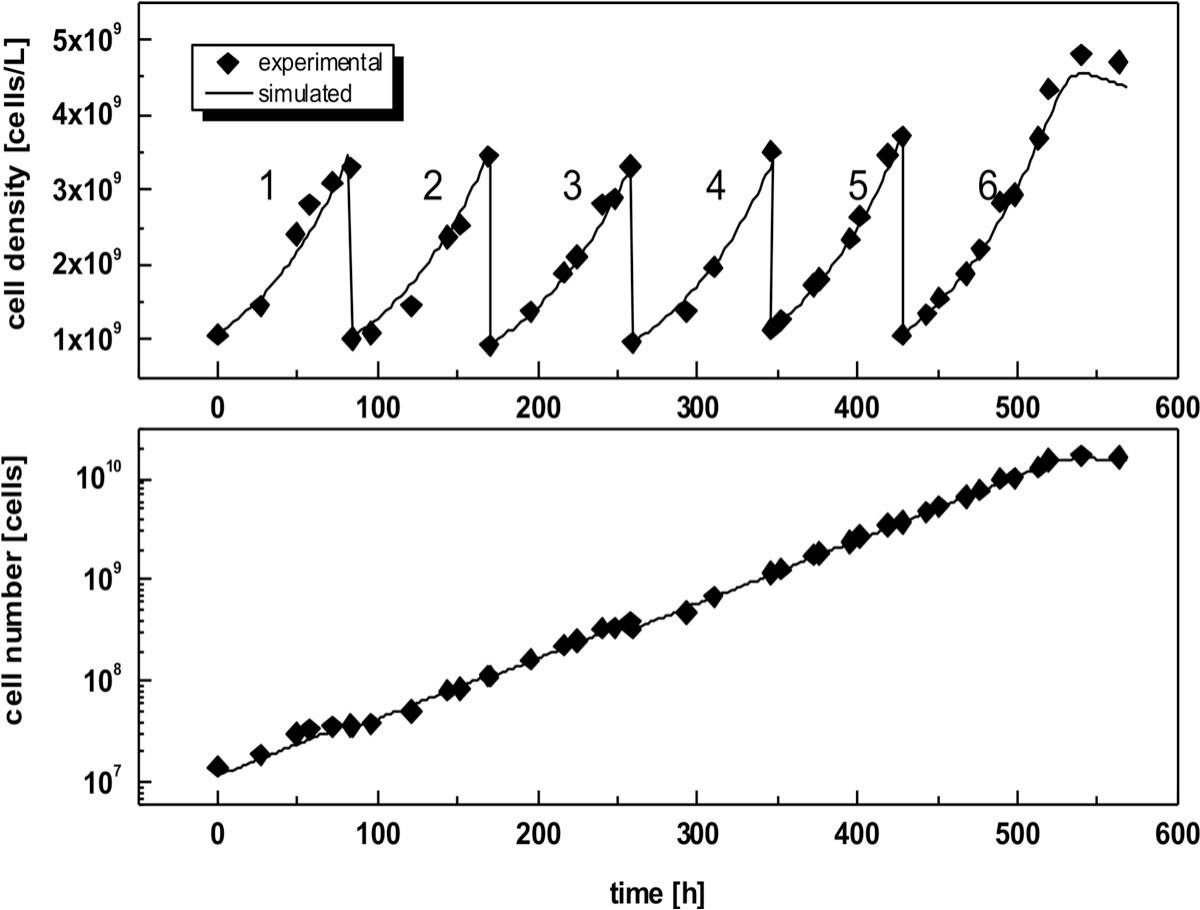
**Time course of simulated and experimentally determined viable cell density and cell number during model based seed from culture tube to lab-scale-bioreactor**. 1: culture tube (0.01 L); 2: shake flask (0.035 L); 3: shake flask (0.13 L), 4: Vario 1000 (medorex, 0.35 L), 5: VSF 2000 (Bioengineering, 1 L); 6: Labfors 5 Cell (Infors, 2.5 L)

The concept was tested successfully up to a 5 L scale for 6 scale-up steps (Figure 1).

## Conclusions

The concept offers a simple and inexpensive strategy for design of the first scale-up steps. The results show that the tool was able to perform a seed train optimization only on the basis of two batches, the underlying model and its parameter identification. This quick optimization led to the same results as the extensive manual optimization carried out in the past.
